# Selective exposure bias predicts views on diversity over time

**DOI:** 10.3758/s13423-022-02167-0

**Published:** 2022-08-29

**Authors:** Jonas De keersmaecker, Katharina Schmid

**Affiliations:** Universitat Ramon Llull, Esade Business School, Barcelona, Spain

**Keywords:** Selective exposure bias, Diversity beliefs, Cognitive bias, Intergroup attitudes

## Abstract

Despite growing diversity, many individuals do not support it, posing a challenge to the successful functioning of societies, institutions, and organizations. We investigated the role of the selective exposure bias on diversity beliefs. In a large-scale nationally representative Spanish sample (*N* = 2,297), we conducted a time-lagged experiment with two time points 5 months apart in which we offered participants a monetary incentive to (allegedly) read attitude contradictory versus conforming information about societal support for refugees. The selective exposure bias asymmetrically predicted future diversity beliefs. Among individuals with a positive intergroup orientation, the selective exposure bias did not predict future diversity beliefs. However, among individuals with a negative intergroup orientation, the selective exposure bias predicted lower pro-diversity beliefs over time, over and above initial pro-diversity beliefs and ideological dispositions. These findings suggest that the absence of pro-diversity beliefs partly originates from a cognitive bias, holding critical implications for policymakers seeking to improve intergroup relations.

Diversity initiatives are at the forefront of many societal, institutional, and organizational efforts to foster inclusion and success (Starck et al., 2021). Yet, despite these proclaimed efforts, across the world, there are many who do not value diversity (Silver et al., 2020), posing an obstacle to equality, positive intergroup relations, and the successful functioning of societies and organizations (Kauff et al., 2021). Prior research has sought to uncover the antecedents of diversity beliefs, showing, for example, that having contact with people from a different ethnic background (Homan, 2019) or people’s ideological orientations (Kauff et al., 2013) are related to diversity beliefs. However, one aspect that has been largely overlooked to date is whether there are any constraints in the development of pro-diversity beliefs that underly the epistemic process. In particular, we argue that individuals’ diversity beliefs are shaped by the selective exposure bias.

People purposefully select information, and avoid exposure to information, in such a manner that one’s attitudes, beliefs, and behaviors are supported rather than challenged (Festinger, 1957). Since early experimental demonstrations of this motivation towards selective exposure (e.g., Adams, 1961), significant progress has been made in understanding who is biased (e.g., Knobloch-Westerwick et al., 2020), when (e.g., Jonas et al., 2001; Lüeders et al., 2019), and why (Metzger et al., 2020). It has been shown that the selective exposure bias occurs in various domains but is stronger for political issues than for others (for a meta-analytic review, see Hart et al., 2009), and, at least in the U.S. and Canada, is equally pronounced among left-wing and right-wing individuals (Frimer et al., 2017). In the context of politics, the avoidance of contradictory ideological information is considered as a defensive mechanism driven by a motivation to avoid cognitive dissonance and maintain a shared reality with significant others (Frimer et al., 2017) as well as a distrust in attitude contradictory news sources (Metzger et al., 2020).

Scholars have argued that the selective exposure bias has the potential to instill ideological extremism and intergroup conflict (Lilienfeld et al., 2009). However, experimental research examining this assertion is scarce. Given the bulk of research focusing on the mere presence or absence of the selective exposure bias (e.g., Barberá et al., 2015), it is surprising how little is known about whether this motivational tendency actually matters for future beliefs and behaviors. Experimental studies typically consider the selective exposure bias as an outcome rather than predictor of future cognitions, and studies that investigated the effects of the selective exposure bias are mostly cross-sectional (e.g., Lüeders et al., 2019) and thus not able to provide evidence about the direction of effects. Furthermore, experiments examining the effects of the selective exposure bias typically ask participants their opinion about an issue, subsequently providing the opportunity to select and read attitude confirming or contradictory information. Yet this classical paradigm of the selective exposure bias is not able to disentangle the effect of (i) one’s motivation for selective exposure and (ii) the actual content of the information provided in the experimental design. Indeed, this paradigm (implicitly) relies on the strong yet untested assumption that the content of the experimentally provided information, its availability, and its consumption are ecologically valid.

Here, we investigated the effect of the selective exposure bias on diversity beliefs, over time. Theoretically, the interaction pattern between one’s intergroup orientations and the selective exposure bias on future diversity beliefs can take different forms. There is consensus that intergroup orientations are not completely fixed and at least partly stem from direct and indirect intergroup experiences (Christ et al., 2014). Therefore, it can be argued that both positive and negative diversity beliefs can partly stem from selective exposure; among people with a negative intergroup orientation, the selective exposure bias should relate negatively to pro-diversity beliefs over time, and among people with a positive intergroup orientation, the selective exposure bias should relate positively to pro-diversity beliefs over time. On the other hand, negative events and information typically have a stronger impact on attitudes than those of positive valence (Baumeister et al., 2001), a phenomenon that has also been repeatedly demonstrated in the context of intergroup attitudes (Barlow et al., 2012). Therefore, it can also be argued that the selective exposure bias has a stronger potential to instill “biased negative diversity beliefs” than “biased positive diversity beliefs.”

Our study tested the predictive value of the selective exposure bias on future diversity beliefs in a longitudinal experiment with two time points spaced 5 months apart using a large representative Spanish sample.

## Method

### Participants

Data collection was subcontracted to a professional survey organization Netquest. Data were collected at two time points approximately 5 months apart—Time 1 (T1): *N* = 2,297; Time 2 (T2): *N* = 2,029. We used quota sampling based on national representative distributions of gender, age, geographical region, and social class (based on a formula that takes into account type of occupation, education level, employment status, household size, and household income) to recruit Spanish citizens whose both parents were born in Spain (*M*_age_ = 49.64 years, *SD* = 16.16; 49% male, 51% female). Exact sample size was determined by project funding. The effect of key interest was the interaction effect between intergroup orientation and the selective exposure bias. Given the explorative research question, it was difficult to estimate the expected means (*M*s), standard deviations (*SD*s), and correlations prior to data collection. A power simulation revealed that this sample provides more than .99% power to detect an interaction effect of β = .10.

### Procedure and measures

After providing online informed consent at T1, participants responded to a three-item measure of pro-diversity beliefs (Kauff et al., 2019; *M* = 5.33, *SD* = 1.39, Cronbach’s α = .93), as well as eight-item measures of right-wing authoritarianism (RWA; based on Altemeyer, 1981; *M* = 3.34, *SD* = 0.97, Cronbach’s α = .68) and social dominance orientation (SDO; based on Pratto et al., 1994; *M* = 2.55, *SD* = 1.02, Cronbach’s α = .77), respectively, all on 7-point Likert scales. Subsequently, we measured the selective exposure bias (based on an adapted procedure from Frimer et al., 2017). First, we asked participants whether they were in favor of or against increasing Spanish support to help refugees coming from North Africa. Next, we assigned participants based on their given answer; participants *in favor of [against]* increasing support were informed that they could win 10 EUR by reading eight arguments *against [in favor of]* investing more resources and answering a question about each argument at T2, or alternatively, to read eight arguments and respond to accompanying questions *in favor of [against]* investing more resources at T2 for a potential cash prize of 7 EUR. Hence, participants were given two options: (a) to read and comment on counterattitudinal arguments to enter a lottery to win 10 EUR, or (b) to read and comment attitude-confirming arguments to enter a lottery to win 7 EUR. The selective exposure bias is operationalized as the willingness to give up the economically maximizing choice in order to read belief confirming opinions. Participants were not actually presented arguments in favor of or against helping refugees.

After providing online informed consent at T2, we measured participants’ diversity beliefs with the same measure (*M* = 5.04, *SD* = 1.51, Cronbach’s α = .95) as at T1. The study is part of a larger project that was approved by the institutional review board. Only the measures relevant for the current investigation are outlined here. Data and code are available online (https://osf.io/wefmk/).

## Results

We found that 53.5% of the participants were in favor of increasing national support for refugees, whereas 46.5% of the participants were against it. In total, 58.6% of the participants showed the selective exposure bias. Among those in favor of supporting refugees, the selective exposure bias (67.7%) was significantly larger than among those against supporting refugees (48%), test of independence, χ^2^(1) = 90.24, *p* < .001.

To examine whether the selective exposure bias predicted diversity beliefs over time, we ran a linear model in which diversity beliefs at T2 were regressed on one’s opinion about helping refugees (against vs. in favor of) at T1, selective exposure bias (no bias vs bias) at T1, and the interaction term between opinion and selective exposure bias, as well as on diversity beliefs at T1 to control for baseline diversity beliefs.

**Fig. 1:**
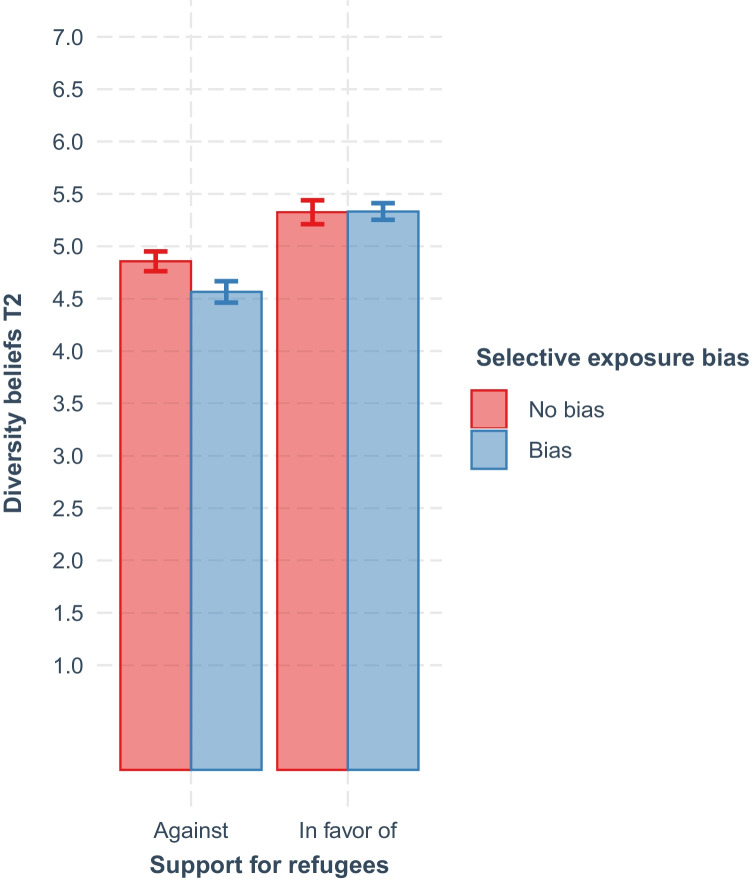
The effects of support for refugees and the selective exposure bias at T1 on diversity beliefs at T2, controlled for diversity beliefs at T1. *Note.* Error bars represent 95% confidence intervals

As expected, results indicated that one’s opinion about helping refugees (*b* = 0.47, *SE* = 0.08, CI_95%_ [0.32, 0.62], *p* < .001) and diversity beliefs at T1 (*b* = 0.64, *SE* = 0.02, CI_95%_ [0.61, 0.68], *p* < .001) predicted diversity beliefs at T2. Critically, the selective exposure bias (*b* = −0.29, *SE* = 0.07, CI_95%_ [−0.43, -0.16], *p* < .001), and the Selective Exposure Bias × Opinion interaction (*b* = 0.30, *SE* = 0.10, CI_95%_ [0.11, 0.49], *p* = .002) also predicted diversity beliefs at T2. This interaction pattern is visualized in Fig. 1; among participants in favor of increasing resources to help refugees, the selective exposure bias did not relate to more favorable diversity beliefs, contrast of estimated marginal means with Bonferroni correction: *t*(2024) = 0.10, *p* = .999. However, among participants who were against increasing resources to help refugees, the selective exposure bias predicted less favorable diversity beliefs, contrast of estimated marginal means with Bonferroni correction: *t*(2024) = 4.23, *p* < .001.

To examine the possibility that the effect of the selective exposure bias on future diversity beliefs was driven by potential differences in (extreme) ideological dispositions, we ran the same model but additionally included two key ideological measures in predicting intergroup outcomes as covariates: RWA and SDO (Duckitt, 2001). RWA taps into a deference for socially conservative norms and social control (Altemeyer, 1981), and SDO captures the degree of preference for inequality among social groups (Pratto et al., 1994). Results revealed that in addition to diversity beliefs at T1 (*b* = 0.58, *SE* = 0.02, CI_95%_ [0.54, 0.62], *p* < .001) and one’s opinion about helping refugees (*b* = 0.35, *SE* = 0.08, CI_95%_ [0.20, 0.50], *p* < .001), RWA (*b* = −0.20, *SE* = 0.03, CI_95%_ [−0.26, −0.14], *p* < .001) and SDO (*b* = −0.06, *SE* = 0.03, CI_95%_ [−0.12, −0.01], *p* = .029) negatively predicted pro-diversity beliefs at T2. Importantly, the selective exposure bias (*b* = −0.22, *SE* = 0.07, CI_95%_ [−0.35, −0.09], *p* < .001), and its interaction with one’s opinion about helping refugees were again significant (*b* = 0.23, *SE* = 0.10, CI_95%_ [0.04, 0.42], *p* = .016). In fact, although the selective exposure bias was larger among the group of individuals who supported versus opposed the idea of increasing resources to support refugees, additional analyses revealed that the presence of the selective exposure bias was not significantly related to individual differences in ideology. Specifically, binomial regressions revealed no evidence for a predictive role of RWA (*b* = 0.03, *SE* = 0.04, CI_95%_ [−0.06, 0.11], *p* = .530), and SDO (*b* = −0.04, *SE* = 0.04, CI_95%_ [−0.13, 0.04], *p* = .294) on the decision to read attitude conforming over contradictory information.

## Discussion

Our study showed that individuals’ views on diversity are shaped by the selective exposure bias. In our experiment, the bias was larger among individuals who supported versus opposed the idea of increasing resources to support refugees. More critically, the selective exposure bias asymmetrically predicted future diversity beliefs. The selective exposure bias among individuals with a positive intergroup orientation towards supporting refugees did not relate to more favorable diversity beliefs over time. In contrast, the selective exposure bias among individuals with a negative intergroup orientation towards supporting refugees predicted less favorable future diversity beliefs, above and beyond initial diversity beliefs. An additional analysis with the inclusion of ideological measures indicated that this effect of the selective exposure bias was not driven by the endorsement of more extreme right-wing attitudes. Hence, the present study suggests that negative opinions about diversity might partly originate from one’s bias to avoid positive information about diversity over time.

There is an ongoing debate in the social sciences whether or not cognitive biases and motivated reasoning are equally pronounced among right-wing and left-wing individuals (see, e.g., Baron & Jost, 2019, versus Ditto et al., 2019). The present results highlight the importance for social scientists to move towards understanding the potential *consequences* of cognitive biases for political attitudes, and to go beyond comparing the mere presence or manifestation of biases between right-wing and left-wing individuals. As our research shows, the relative absence—but not presence—of pro-diversity beliefs partly originates from the selective exposure bias. This finding suggests that not everyone’s biases have a similar (negative) impact on one’s attitudes and behaviors. Thus, the question of who is biased—although important—constitutes only a small piece of a complex puzzle in our understanding of ideological attitudes.

A strength of the current investigation is that the experiment consisted of two time points several months apart in which we did not present participants with attitude confirming or contradictory information. By not providing participants with actual arguments, we were able to focus on the *motivation* effect towards the selective exposure bias rather than the effect of *consuming* selective information.

Notwithstanding the strengths of our paper, our methodology is not without its limitations. For one, we did not examine antecedents of the selective exposure bias, and whether they were the same for individuals who were in favor of or against increasing resources to help refugees (cf. Frimer et al., 2017). Additionally, the employed design also did not allow us to determine whether the selective exposure bias was driven by a motivation to seek out attitude confirming information or a motivation to avoid attitude contradictory information, or a combination of both. An interesting avenue for future research will be to disentangle these motivations. Furthermore, we measured the selective exposure bias in a domain most relevant for diversity beliefs (i.e., national support for refugees). Intergroup orientations are partly rooted in fundamental cognitive processes (Crisp & Meleady, 2012). For example, previous research demonstrated that domain independent cognitive factors such as a general need for cognitive closure and cognitive ability predict intergroup relations (De keersmaecker et al., 2018). Therefore, future research might examine whether a general tendency for selective exposure bias also predicts diversity beliefs over time.

Providing positive intergroup experiences is a key strategy to increase support for diversity and improve intergroup relations, especially among individuals who hold a negative intergroup orientation to begin with (Hodson, 2011). However, people’s tendency to display a selective exposure bias challenges efforts to the development of pro-diversity beliefs. The present investigation has shown that even at the risk of incurring known personal costs, people can be motivated to affirm their beliefs, with critical ramifications for their future beliefs. We therefore recommend that pro-diversity initiatives should be tailored in such a way to avoid positive intergroup experiences being optional, but to make them inevitable.

## Data Availability

Data are available: https://osf.io/wefmk/.
